# Association of rotator cuff degeneration and scapular anatomy with humeral head migration in rotator cuff arthropathy

**DOI:** 10.1002/jeo2.70219

**Published:** 2025-04-01

**Authors:** Hannes E. Tytgat, Nazanin Daneshvarhasjin, Philippe Debeer, Jean Chaoui, Filip Verhaegen

**Affiliations:** ^1^ Department of Orthopedics UZ Leuven Leuven Belgium; ^2^ Digital Technologies Trauma & Extremities, Stryker Portage Michigan USA

**Keywords:** glenoid version, humeral head migration, rotator cuff arthropathy, rotator cuff atrophy, rotator cuff fatty infiltration, scapular anatomy

## Abstract

**Purpose:**

Rotator cuff tear arthropathy (RCTA) is characterised by humeral head migration (HHM). The exact pathogenesis of HHM is poorly understood, although rotator cuff (RC) failure and scapular anatomy are thought to play an important role. The aim of this study is to investigate the possible association between HHM and the quantitative aspects of scapular anatomy and RC degeneration.

**Methods:**

We analysed computed tomography scans of 43 RCTA patients. RC fatty infiltration (FI) and atrophy, HHM, and both native and pathologic scapular anatomy were quantitatively assessed in three dimensions.

**Results:**

Patients with superior HHM had a significantly higher critical shoulder angle (34° vs. 30°, *p* = 0.009), and FI of the supraspinatus (26% vs. 16%, *p* = 0.025) and infraspinatus (IS) (25% vs. 16%, *p* = 0.038) compared to patients without superior HHM. Patients with posterior HHM had a significantly more retroverted native (mean 10° vs. 6°; *p* = 0.002) and pathologic glenoid (mean 11° vs. 4°; *p* = 0.001) and a higher anterior axis length (mean 40 mm vs. 37 mm; *p* = 0.001) compared to patients without anteroposterior HHM. Multivariate regression analysis showed that the native glenoid version, anterior axis length and the volume (Vol) of IS divided by subscapularis (*p* = 0.01) were independent predictors of the magnitude of anteroposterior HHM, together explaining 41% of its variance.

**Conclusion:**

In RCTA, degeneration of the posterosuperior RC and acromion morphology seems to be associated with superior HHM, while in the glenoid version, the rotational alignment of the coracoacromial complex and an imbalance in FI and muscle Vol in the transverse force couple seems to be associated with anteroposterior HHM.

**Level of Evidence:**

Level III.

**Abbreviations**
2D: two‐dimensional
CSA: critical shoulder angle
FI: fatty infiltration
FV: functional volume
HHM: humeral head migration
IS: infraspinatus
LAA: lateral acromial angle
PAS: posterior acromial slope
RC: rotator cuff
RCTA: rotator cuff tear arthropathy
SLD: subluxation distance
SLD‐AP: SLD anteroposterior
SLD‐SI: SLD superoinferior
SS: supraspinatus
Ssc: subscapularis
Vol: volume

## INTRODUCTION

Rotator cuff tear arthropathy (RCTA) is one of the most frequent types of shoulder arthropathy [23]. It represents a broad spectrum of pathology in which three critical factors are present: rotator cuff (RC) failure, humeral head migration (HHM) and glenohumeral joint degeneration [27]. The exact pathogenesis of RCTA remains unknown, but the hallmark is the failure of the RC. In the normal glenohumeral joint, the rotator cuff functions as a dynamic stabiliser by compressing the humeral head against the concave surface of the glenoid [19]. When those compressive forces fail to counteract the superior pull of the deltoid muscle, the humeral head can migrate superiorly, leading to the destruction of the humeral head and scapular bone [15, 18, 28]. While the superior HHM is most apparent and is incorporated in the definition of RCTA, there is also an important anteroposterior component to this HHM, but knowledge about its pathogenesis is limited [1, 6, 9]. A hypothesis is that posterior migration originates from the absence of the infraspinatus (IS) muscle combined with the posteriorly directed force of the latissimus dorsi, and similarly that anterior migration could occur due to the anterior pull of the pectoralis major in the absence of the subscapularis (Ssc) muscle [9, 13].

Previous research has investigated possible determinants of the quantitative aspects of HHM in RCTA, such as native scapular anatomy (e.g., native glenoid retroversion, acromion morphology and rotational alignment of the coracoacromial complex) [8, 9, 24], pathologic scapular anatomy [10, 14, 31] and RC degeneration [1, 15, 28]. RC degeneration can be characterised by fatty infiltration (FI) and muscle atrophy, which strongly correlate with muscle strength [17, 32]. Evidence of an association between HHM and RC degeneration is conflicting in shoulder OA [7, 29, 37], and even more limited in RCTA. In RCTA patients with posterosuperior glenoid erosion, FI was found to be much more pronounced in the posterosuperior RC compared to the Ssc [1]. Although an association between pathologic scapular anatomy and quantitative aspects of HHM can be intuitively expected, most studies could not find a correlation [10, 14, 36]. This lack of clear association of both pathologic scapular anatomy and RC degeneration with HHM is possibly due to methodological problems in the quantification of these parameters. Most studies used two‐dimensional (2D) measurement techniques for quantification of HHM, RC degeneration and scapular anatomy, which has been shown to be less reliable [25, 31, 38]. In contrast, three‐dimensional (3D) quantitative measurement techniques can improve the accuracy [3, 20, 31].

The aim of this study is to investigate the possible association between HHM on the one hand and the quantitative aspects of scapular anatomy and RC degeneration on the other hand. We hypothesise that (1) quantitative aspects of pathologic scapular anatomy and RC degeneration are associated with the amount of HHM, and more specifically (2), that the transverse force couple imbalance, together with glenoid version are associated with the amount of anteroposterior HHM.

## MATERIALS AND METHODS

This retrospective case–control study was approved by the ethical committee of the University Hospitals Leuven (S58348). From a mixed computed tomography (CT) scan data set of patients undergoing reversed total shoulder arthroplasty and patients without radiographic signs of HHM or arthropathy (control group) as judged by an experienced shoulder surgeon (Filip Verhaegen), 64 patients with RCTA and 49 control patients were included based on the availability of the entire scapula and proximal humerus on the CT scan images, as previously reported in another study [9].

### Quantification of rotator cuff degeneration

**Figure 1:**
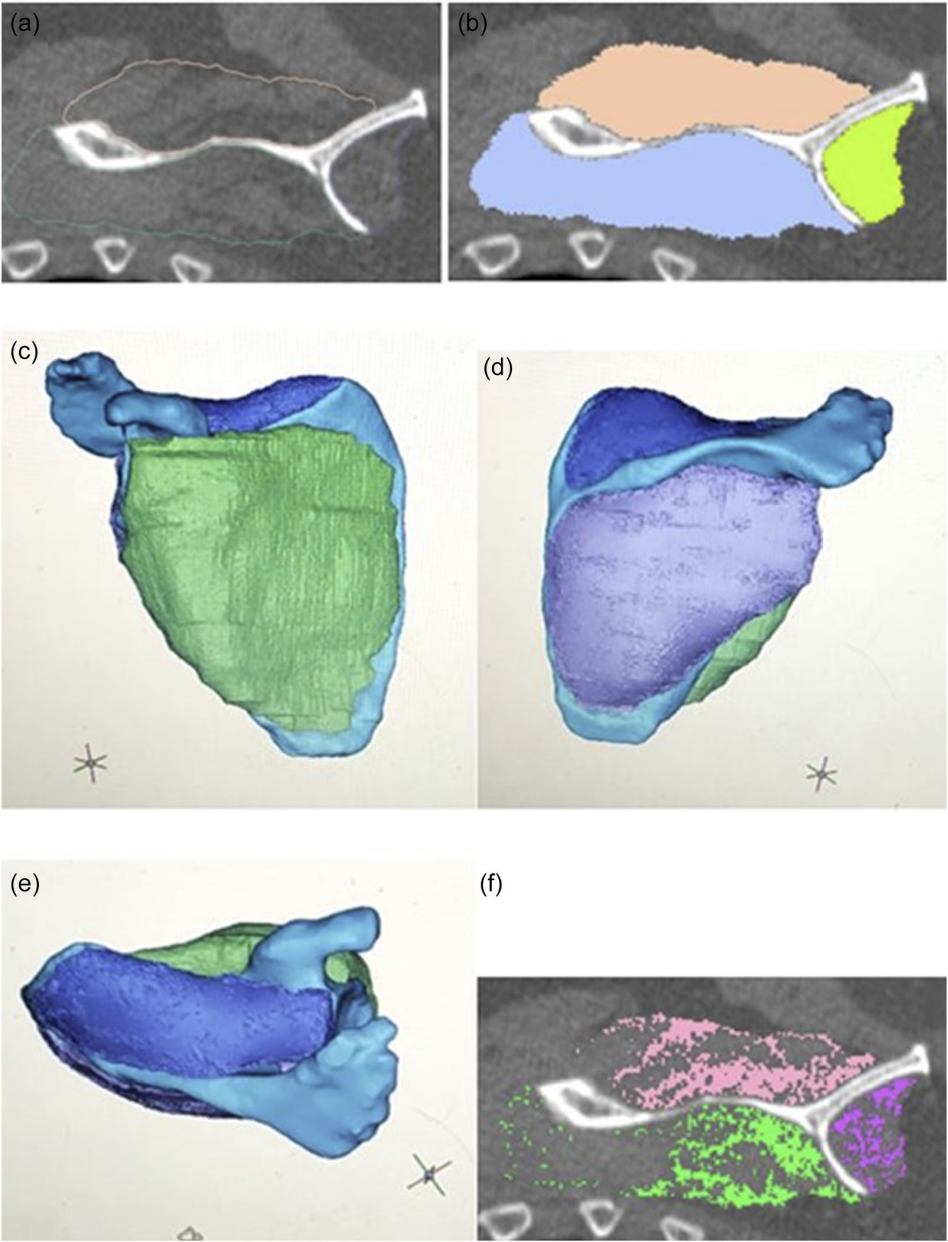
Quantification of rotator cuff degeneration. (a) Example of sagittal slice with delineated supraspinatus (blue), infraspinatus (orange) and subscapularis (green). (b) Example of sagittal slice depicting total muscle volume. (c–e) Example of 3D total muscle volume of RC muscles. (f) Sagittal slice depicting fatty tissue (<−29 HU) within each RC muscle. 3D, three‐dimensional; HU, Hounsfield units; RC, rotator cuff.

The soft‐tissue Digital Imaging and Communications in Medicine (DICOM) images for each patient were uploaded into Mimics software (version 22.0; Materialise®). The scapular bone and the RC muscles supraspinatus (SS), Ssc and IS combined with teres minor were manually segmented by delineating the borders in the coronal, sagittal and axial plane [3, 38] (Figure 1a–e).

To quantify the RC muscle atrophy, the total muscle volume (Vol) of the different RC muscles was normalised to the patient's scapular bone Vol. To quantify the RC muscle FI, the fat Vol within each of the RC muscle Vols was determined based on a predefined threshold for fat (<−29 Hounsfield units [HU]) [30, 38] (Figure 1f). The obtained fat Vol was divided by the total muscle Vol, creating FI percentage within each cuff muscle. Finally, the functional volume (FV), representing the actual amount of RC muscle fibres, was calculated by subtracting the fat Volfrom from the total muscle Vol and then normalising it to the scapular bone Vol. The transverse force couple was defined as the obtained values for Vol, FI and FV of IS divided by Ssc [16], although Vol and FI are not the only determinants of force (e.g., fibre to muscle length ratio, fibre recruitment and angle of contraction).

### Quantification of HHM

HHM was quantified with a previously reported and validated statistical shape model‐based methodology [35]. In short, the native glenoid centre point and humeral head centre point, defined as the centre of mass of, respectively, all reconstructed glenoid surface points and all points of the proximal humerus are determined. The subluxation distance (SLD) is defined as the distance between the glenoid centre point and the projection of the humeral head centre point on the reconstructed glenoid plane. In addition, SLD is further divided into an anteroposterior (SLD‐AP) and a superoinferior (SLD‐SI) component based on the superior glenoid axis, which is the projection of the superior scapular axis on the reconstructed glenoid plane [9, 34]. A cut‐off value for confirming HHM was based on the ‘mean + 2 × standard deviation’ of SLD in the control group. RCTA patients were grouped as ‘RCTA with HHM (Group 1)’ and ‘RCTA without HHM (Group 2)’. Using the same methodology, a further subdivision was made in the anteroposterior and superoinferior direction, creating ‘RCTA with posterior migration (Group 3)’, ‘RCTA with anterior migration (Group 4)’, ‘RCTA without anteroposterior migration (Group 5)’, ‘RCTA with superior migration (Group 6)’ and ‘RCTA without superior migration (Group 7)’. The mean values and standard deviation of the control group and their resulting cut‐off values can be found in Appendix S1.

### Quantification of scapular anatomy

The parameters of the native scapular anatomy were automatically quantified with a previously published and validated 3D Statistical shape model‐based methodology: native glenoid version and inclination, scapular offset, critical shoulder angle (CSA), lateral acromial angle (LAA), posterior acromial slope (PAS) and parameters describing the rotational alignment of the coracoacromial complex [22]. The pathologic scapular anatomy in terms of glenoid version and inclination was automatically quantified using the commercially available Glenosys software (Imascap®) [4].

### Statistics

A priori power analysis was not performed due to the primarily explorative nature of this study. Descriptive statistics of RC degeneration, HHM, and scapular anatomy were performed for all groups. Normality of the variables was assessed by visual inspection of *Q*–*Q* plots and normality tests (Kolmogorov–Smirnov and Shapiro–Wilk). Statistical significance was considered at *p* < 0.05. Because of the non‐normality of some parameters, the scapular anatomy and RC degeneration of Group 1 versus Group 2, and Group 6 versus Group 7 were evaluated with the Mann–Whitney *U* test, while the Kruskall–Wallis test was used for comparison between Groups 3 and 5. When the results were significant, pairwise (post hoc) comparisons were performed using the Mann–Whitney *U* test. To correct for Type I errors, a Bonferroni correction was applied, setting the significance level at *p* < 0.02. A stepwise multivariate regression analysis was performed to analyse the influence of the variables on the magnitude of SLD, SLD‐AP and SLD‐SI. In order to prevent overfitting of the models, we only included the parameters of the native scapular anatomy that were previously found to be significant determinants of HHM (LAA and CSA for SLD; native version and LAA for SLD‐AP; scapular offset for SLD‐SI) [9], and the other variables that were found to be significantly correlated after a univariate linear regression analysis (*p* < 0.05; *r* < 0.33 = weak; 0.33 < *r* > 0.66 = moderate; *r* > 0.66 = strong).

## RESULTS

Of the initial data set, 21 patients were excluded due to the absence of soft tissue windows on the available CT scan images, leaving a total of 43 patients for the RCTA group. Of this group, three patients were excluded from the pathologic scapular anatomy quantification because of the incompatibility of the CT scan images with the Glenosys software (Imascap®).

### Rotator cuff degeneration

FI was more pronounced in the SS (mean 24%) and IS (mean 23%) compared to the Ssc (mean 10%, *p* < 0.001). The FV(Ssc) was the highest (1.15, *p* < 0.001). With regard to the transverse force couple, IS had a lower Vol and more FI than Ssc (mean Vol(IS/Ssc) 0.81; mean FI(IS/Ssc) 2.15) (Table 1).

**Table 1:** Quantification of rotator cuff degeneration in terms of volume (Vol), fatty infiltration (FI) and functional volume (FV).

		Mean (±SD)	*p* value
Vol	SS	0.36 (±0.06)	<0.001
	IS	1.09 (±0.17)	<0.001
	Ssc	1.32 (±0.24)	<0.001
	IS/Ssc^a^	0.81 (±0.15)	
FI	SS	0.24 (±0.12)	<0.001*
	IS	0.23 (±0.13)	<0.001*
	Ssc^a^	0.10 (±0.12)	<0.001
	IS/Ssc^a^	2.15 (±25.64)	
FV	SS	0.27 (±0.07)	<0.001
	IS	0.83 (±0.17)	<0.001
	Ssc	1.15 (±0.24)	<0.001
	IS/Ssc	0.76 (±0.22)	

Abbreviations: IS, infraspinatus; SS, supraspinatus; Ssc, subscapularis.

^a^ Abnormally distributed with median value instead of mean.

* *p* value between FI(SS) and FI(IS) is 0.504.

### Quantification of HHM and scapular anatomy

**Figure 2:**
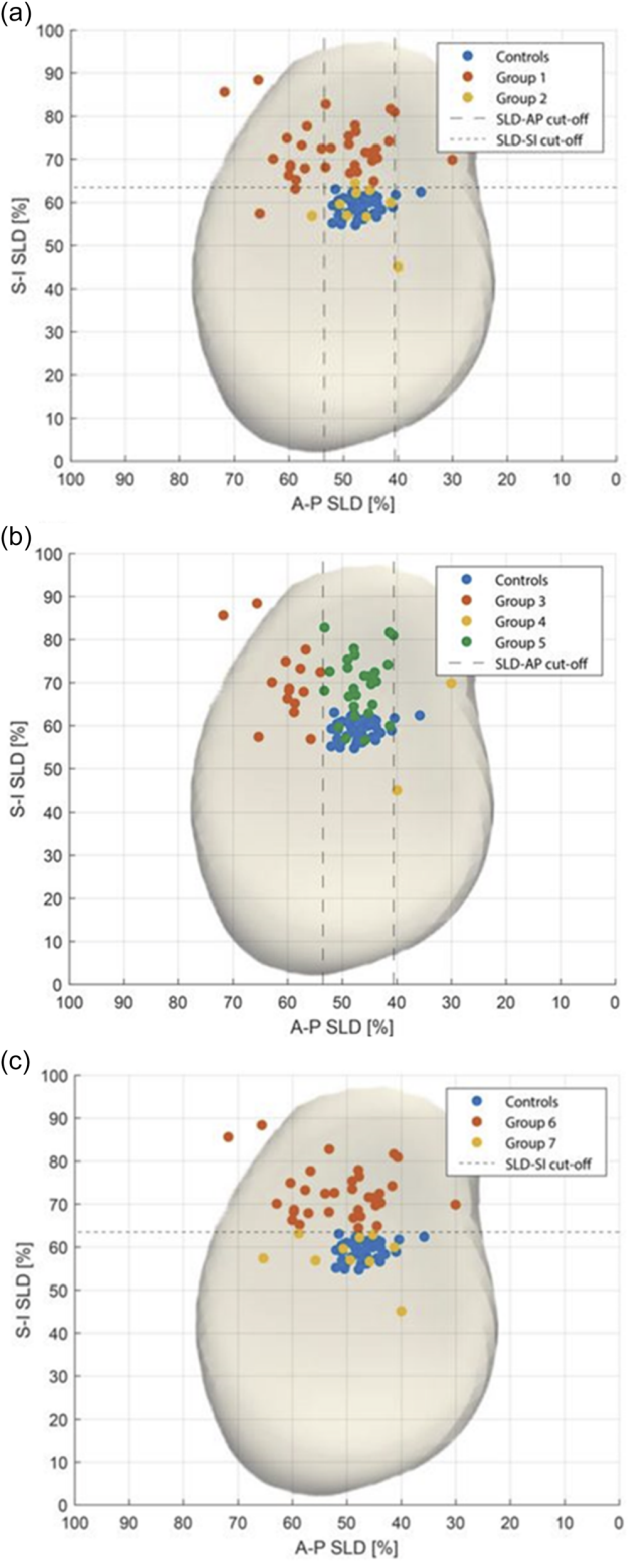
Polar plots from different subgroups. (a) Polar plot from control group, Group 1 (RCTA with HHM) and Group 2 (RCTA without HHM). (b) Polar plot from control group, Group 3 (RCTA with posterior HHM), Group 4 (RCTA with anterior HHM) and Group 5 (RCTA without AP HHM). (c) Polar plot from control group, Group 6 (RCTA with superior HHM) and Group 7 (RCTA without superior HHM). AP, anteroposterior; HHM, humeral head migration; RCTA, rotator cuff tear arthropathy.

The mean SLD of the RCTA patients was 71% (SD ± 8%), and the mean SLD‐AP and SLD‐SI was 51% (SD ± 8%) and 69% (SD ± 9%), respectively. The mean SLD in Group 1 (*n* = 34) was 74% (SD 7%), as compared to 61% (SD3%) in Group 2 (*n* = 9, *p* = 0.001) (Figure 2a). As for anteroposterior HHM, Group 3 (*n* = 15) had a significantly higher SLD‐AP of 60% (SD ± 5%, *p* = 0.001), compared to Group 4 (*n* = 2; SLD‐AP = 35% ± SD 7%) and Group 5 (*n* = 26; SLD‐AP = 47% ± SD 4%) (Figure 2b). Finally, Group 6 (*n* = 33) and Group 7 (*n* = 10) had a mean SLD‐SI of 73 ± 6% and 58 ± 5% (*p* = 0.001) (Figure 2c), respectively.

The parameters of the native and pathologic scapula are shown in Table 2. The pathologic glenoid had a mean retroversion of 7° (SD ± 7°) and a mean superior inclination of 12° (SD ± 6°).

**Table 2:** Parameters of the native and pathologic scapula of all RCTA patients (mean ± SD).

	Mean (±SD)
Native inclination	8° (±4°)
Native version	7° (±4°)
Critical shoulder angle^a^	33° (±4°)
Posterior acromial slope	64° (±8°)
Lateral acromial angle	94° (±5°)
Scapular offset	102 mm (±6 mm)
Coracoid‐scapula angle^a^	63° (±6°)
Anterior acromion‐scapular plane angle	−1° (±9°)
Posterior acromion‐scapular plane angle	56° (±8°)
Total fulcrum axis length	70 mm (±5 mm)
Fulcrum axis ratio^a^	45% (±4%)
Anterior axis length^a^	38 mm (±3 mm)
Posterior axis length	32 mm (±4 mm)
Glenoid translation	7 mm (± 3 mm)
Pathologic inclination	12° (±6°)
Pathologic version	7° (±7°)

Abbreviations: RCTA, rotator cuff tear arthropathy; SD, standard deviation.

^a^ Abnormally distributed with median value instead of mean.

### Association of HHM with RC degeneration and scapular anatomy

Statistical analysis showed a significantly higher CSA (33° vs. 31°, *p* = 0.039) and LAA (95° vs. 91°, *p* = 0.034) for Group 1 in comparison to Group 2. In addition, the SS showed significantly more FI (26% vs. 16%, *p* = 0.029) for Group 1 (Table 3). The other parameters did not differ significantly between Groups 1 and 2. Univariate regression analysis showed a moderate correlation of SLD with FI(SS) (*r* = 0.40; *p* = 0.008), FI(IS) (*r* = 0.39; *p* = 0.015) and FV(IS) (*r* = −0.34; *p* = 0.025). Multiple regression analysis (including CSA, LAA, FI(SS), FI(IS) and FV(IS)) showed that FI(SS) was an independent significant predictor on the magnitude of SLD (*p* = 0.008) (see Appendix S2). Variance of SLD can be explained by FI(SS) for 14%. Regression predicts every increase of FI(SS) with 10% to be associated with an increase of SLD of 2.6% (95% confidence interval [CI]: 0.7–4.4).

**Table 3:** Parameters of RC degeneration and scapular anatomy for Groups 1 and 2 (mean ± SD).

	Group 1: with HHM (*n* = 34)	Group 2: without HHM (*n* = 9)	*p* value
Native inclination	8° (±4°)	6° (±5°)	0.199
Native version	8° (±5°)	6° (±3°)	0.339
**Critical shoulder angle^a^ **	**33° (±4°)**	**31° (±3°)**	**0.039**
Posterior acromial slope	64° (±9°)	65° (±7°)	0.881
**Lateral acromial angle**	**95° (±5°)**	**91° (±5°)**	**0.034**
Scapular offset	103 mm (±6 mm)	102 mm (±6 mm)	0.676
Coracoid‐scapula angle^a^	64° (±5°)	60° (±7°)	0.31
Anterior acromion‐scapular plane angle	−1° (±9°)	0° (±9°)	0.591
Posterior acromion‐scapular plane angle	56° (±8°)	56° (±8°)	0.788
Total fulcrum axis length	70 mm (±6 mm)	71 mm (±4 mm)	0.244
Fulcrum axis ratio^a^	46% (±4%)	45% (±5%)	0.55
Anterior axis length^a^	38 mm (±3 mm)	37 mm (±4 mm)	0.834
Posterior axis length	32 mm (±5 mm)	33 mm (±4 mm)	0.42
Glenoid translation	6 mm (±6 mm)	7 mm (±2 mm)	0.455
Pathologic inclination	12° (±6°)	9° (±6°)	0.144
Pathologic version	7° (±7°)	6° (±5°)	0.773
Vol(SS)	0.36 (±0.07)	0.35 (±0.05)	0.976
**FI(SS)**	**0.26 ( ± 0.13)**	**0.16 (** ± **0.08)**	**0.029**
Vol(IS)	1.09 (±0.17)	1.08 (±0.19)	0.698
FI(IS)	0.24 (±0.14)	0.19 (±0.12)	0.325
Vol(Ssc)	1.31 (±0.26)	1.38 (±0.17)	0.339
FI(Ssc)^a^	0.10 (±0.13)	0.07 (±0.06)	0.199
Vol(IS/Ssc)^a^	0.81 (±0.15)	0.78 (±0.11)	0.355
FI(IS/Ssc)^a^	1.83 (±28.83)	2.67 (±1.97)	0.403
FV(SS)	0.27 (±0.07)	0.30 (±0.06)	0.152
FV(IS)	0.82 (±0.15)	0.89 (±0.24)	0.21
FV(Ssc)	1.11 (±0.25)	1.27 (±0.20)	0.064
FV(IS/Ssc)	0.77 (±0.23)	0.70 (±0.16)	0.571

*Note*: Underline bold values are statistical significant results.

Abbreviations: FI, fatty infiltration; FV, functional volume; IS, infraspinatus; SS, supraspinatus; Ssc, subscapularis; Vol, volume.

^a^ Abnormally distributed with median value instead of mean.

### Association of anteroposterior HHM with RC degeneration and scapular anatomy

Group 3 patients showed a significantly more retroverted native (mean 10° vs. 6°; *p* = 0.002) and pathologic glenoid (mean 11° vs. 4°; *p* = 0.001) and a higher anterior axis length (mean 40 mm vs. 37 mm; *p* = 0.001) compared to Group 5 (Table 4). Furthermore, there was a trend towards a smaller fulcrum axis ratio (44% vs. 47%, *p* = 0.077) and a higher Vol(IS/Ssc) (0.87 vs. 0.78; *p* = 0.17) in Group 3. No statistical significance was found between Group 4 and Group 3 or 5. Univariate regression analysis showed a moderate correlation of SLD‐AP with anterior axis length (*r* = 0.51; *p* < 0.001), pathologic version (*r* = 0.45; *p* = 0.004) and Vol(IS/Ssc) (*r* = 0.33; *p* = 0.03), and a weak correlation with fulcrum axis ratio (*r* = −0.31; *p* = 0.044).

**Table 4:** Parameters of RC degeneration and scapular anatomy for Groups 3, 4 and 5 (mean ± SD).

	Group 3: anterior HHM (*n* = 15)	Group 4: posterior HHM (*n* = 2)	Group 5: no AP HHM (*n* = 26)	*p* value	Group 3 vs. Group 5
Native inclination	9° (±4°)	6° (±4°)	7° (±4°)	0.382	
**Native version**	**10° (±4°)**	**7° (±4°)**	**6° (±4°)**	**0.008**	**0.002**
Critical shoulder angle^a^	32° (±5°)	31° (±8°)	33° (±4°)	0.92	
Posterior acromial slope	65° (±9°)	60° (±1°)	64° (±8°)	0.725	
Lateral acromial angle	94° (±5°)	92° (±5°)	94° (±6°)	0.738	
Scapular offset	104 mm (±5 mm)	98 mm (±1 mm)	102 mm (±6 mm)	0.332	
Coracoid‐scapula angle^a^	65° (±5°)	57° (±6°)	61° (±5°)	0.11	
Anterior acromion‐scapular plane angle	0° (±9°)	−8° (±3°)	−1° (±10°)	0.486	
Posterior acromion‐scapular plane angle	54° (±10°)	57° (±5°)	57° (±6°)	0.654	
Total fulcrum axis length	72 mm (±6 mm)	68 mm (±3 mm)	69 mm (±5 mm)	0.153	
Fulcrum axis ratio^a^	44% (±5%)	48% (±1%)	47% (±3%)	0.077	
Anterior axis length^a^	**40 mm (±4 mm)**	**35 mm (±1 mm)**	**37 mm (±2 mm)**	**0.001**	**0.001**
Posterior axis length	32 mm (±5 mm)	33 mm (±2 mm)	32 mm (±4 mm)	0.599	
Glenoid translation	6 mm (±3 mm)	6 mm (±mm)	7 mm (±3 mm)	0.354	
Pathologic inclination	12° (±6°)	−1°	12° (±5°)	0.237	
Pathologic version	**11° (±6°)**	**7°**	**4° (±6°)**	**0.003**	**0.001**
Vol(SS)	0.37 (±0.06)	0.35 (±0.06)	0.35 (±0.07)	0.429	
FI(SS)	0.26 (±0.10)	0.23 (±0.26)	0.22 (±0.13)	0.346	
Vol(IS)	1.14 (±0.15)	1.13 (±0.12)	1.05 (±0.13)	0.317	
FI(IS)	0.24 (±0.11)	0.31 (±0.32)	0.22 (±0.13)	0.725	
Vol(Ssc)	1.30 (±0.25)	1.34 (±0.16)	1.33 (±0.24)	0.945	
FI(Ssc)^a^	0.10 (±0.12)	0.26 (±0.11)	0.08 (±0.12)	0.183	
Vol(IS/Ssc)^a^	0.87 (±0.17)	0.85 (±0.19)	0.78 (±0.13)	0.17	
FI(IS/Ssc)^a^	2.01 (±43.29)	1.02 (±0.79)	2.23 (±1.92)	0.4	
FV(SS)	0.28 (±0.06)	0.28 (±0.14)	0.27 (±0.06)	0.993	
FV(IS)	0.86 (±0.13)	0.76 (±0.28)	0.82 (±0.18)	0.768	
FV(Ssc)	1.13 (±0.27)	1.00 (±0.27)	1.17 (±0.23)	0.484	
FV(IS/Ssc)	0.82 (±0.28)	0.75 (±0.08)	0.72 (±0.19)	0.649	

Abbreviations: FI, fatty infiltration; FV, functional volume; IS, infraspinatus; RC, rotator cuff; SS, supraspinatus; Ssc, subscapularis; Vol, volume.

^a^ Abnormally distributed with median value instead of mean.

Multivariate regression analysis (including native and pathologic version, LAA, anterior axis length, Vol(IS/Ssc) and fulcrum axis ratio) showed that native version (*p* = 0.002), native version combined with Vol(IS/Ssc) (*p* = 0.015), and native version combined with Vol(IS/Ssc) and anterior axis length (*p* = 0.01) were independent significant predictors on the magnitude of SLD‐AP (see Appendix S2). Variance of SLD‐AP can be explained by these models as 21%, 31% and 41%, respectively. For the latter combination (native version combined with Vol(IS/Ssc) and anterior axis length), regression predicts every degree increase of native retroversion and every 1 mm increase of anterior axis length to be associated with an increase of SLD‐AP of 1% (95% CI: 0.07%–1.05% and 95% CI: 0.21%–1.43%, respectively). Every increase of Vol(IS/Ssc) with 0.1 is associated with an increase of SLD‐AP of 2% (95% CI: 0.69%–3.35%).

### Association of superoinferior HHM with RC degeneration and scapular anatomy

Group 6 had a significantly higher CSA (34° vs. 30°, *p* = 0.009), FI(SS) (26% vs. 16%, *p* = 0.025) and FI(IS) (25% vs. 16%, *p *= 0.038) compared to Group 7 (Table 5). There was also a trend towards a higher pathologic inclination (12° vs. 8°; *p* = 0.139). Univariate regression analysis showed a moderate correlation of SLD‐SI with pathologic inclination (*r* = 0.34; *p* = 0.03), FI(SS) (*r* = 0.42; *p* = 0.005), FI(IS) (*r* = 0.34; *p* = 0.024) and FV(SS) (*r* = −0.33; *p* = 0.032), and a weak correlation with FV(IS) (*r* = −0.31; *p* = 0.045)

**Table 5:** Parameters of RC degeneration and scapular anatomy for Groups 6 and 7 (mean ± SD).

	Group 6: superior HHM (*n* = 33)	Group 7: no superior HHM (*n* = 10)	*p* value
	Mean	Mean	
Native inclination	8° (±4°)	6° (±5°)	0.262
Native version	7° (±4°)	8° (±5°)	0.646
**Critical shoulder angle^a^ **	**34° (±4°)**	**30° (±3°)**	**0.009**
Posterior acromial slope	64° (±9°)	65° (±6°)	0.931
Lateral acromial angle	95° (±6°)	92° (±5°)	0.135
Scapular offset	103 mm (±6 mm)	102 mm (±6 mm)	0.687
Coracoid‐scapula angle^a^	64° (±5°)	61° (±8°)	0.666
Anterior acromion‐scapular plane angle	−1° (±10°)	−1° (±9°)	0.73
Posterior acromion‐scapular plane angle	56° (±8°)	56° (±8°)	0.646
Total fulcrum axis length	70 mm (±6 mm)	71 mm (±4 mm)	0.287
Fulcrum axis ratio^a^	46% (±4%)	45% (±5%)	0.908
Anterior axis length^a^	38 mm (±3 mm)	37 mm (±4 mm)	0.709
Posterior axis length	32 mm (±5 mm)	32 mm (±4 mm)	0.752
Glenoid translation	7 mm (±3 mm)	6 mm (±3 mm)	0.908
pathologic inclination	12° (±6°)	8° (±7°)	0.139
pathologic version	6° (±7°)	9° (±5°)	0.243
Vol(SS)	0.36 (±0.07)	0.35 (±0.06)	1
**FI(SS)**	**0.26 (** ± **0.12)**	**0.16 (** ± **0.10)**	**0.025**
Vol(IS)	1.10 (±0.17)	1.05 (±0.18)	0.687
**FI(IS)**	**0,25 (** ± **0.14)**	**0,16 (** ± **0.10)**	**0.038**
Vol(Ssc)	1.34 (±0.25)	1.28 (±0.22)	0.585
FI(Ssc)^a^	0.10 (±0.13)	0.06 (±0.08)	0.301
Vol(IS/Ssc)^a^	0.81 (±0.14)	0.82 (±0.17)	1
FI(IS/Ssc)^a^	2.01 (±29.25)	2.48 (±1.96)	0.863
FV(SS)	0.26 (±0.06)	0.30 (±0.07)	0.216
FV(IS)	0.81 (±0.15)	0.90 (±0.23)	0.121
FV(Ssc)	1.14 (±0.24)	1.17 (±0.25)	0.565
FV(IS/Ssc)	0.74 (±0.21)	0.79 (±0.26)	0.546

Abbreviations: FI, fatty infiltration; FV, functional volume; IS, infraspinatus; SS, supraspinatus; Ssc, subscapularis; Vol, volume.

^a^ Abnormally distributed with median value instead of mean.

Multivariate regression analysis of SLD‐SI (including scapular offset, pathologic inclination, FI(SS), FI(IS), FV(SS) and FV(IS)) showed that FV(IS) was an independent significant predictor on the magnitude of SLD‐SI, explaining variance for 17% (see Appendix S2). Regression predicts every decrease of FV(IS) with 0.1 to be associated with an increase of SLD‐SI of 2.5% (95% CI: −4.2 to −0.8).

## DISCUSSION

The most important findings of this study are that degeneration of the posterosuperior RC, pathologic superior glenoid inclination and acromion morphology are associated with superior HHM, while glenoid version, transverse force couple imbalance and the rotational alignment of the coracoacromial complex are associated with anteroposterior HHM.

We found a moderate correlation between FI and FV of the posterosuperior RC and superior HHM. This is in accordance with previous findings, which showed that superior HHM occurs when an RC tear extends from the SS to the IS [15, 18, 28]. We also found that RCTA patients with superior HHM had a significantly higher CSA. A high CSA, which is influenced by the lateral extension of the acromion and the glenoid inclination, has been previously reported to be associated with increased risk and magnitude of RC failure, as well as increased superior HHM [8, 24]. The postulated patho‐mechanism is believed to be twofold, with, on the one hand, an increased compression of the SS tendon and, on the other hand, an increased ascending force vector and decreased compressive force vector of the middle deltoid [24]. When controlling for confounding variables in a multivariate regression analysis, only FV(IS) remained as an independent significant predictor. Interestingly, scapular anatomy was not withheld as an independent predictor. This could suggest that superior HHM is mostly soft tissue driven.

Regarding the anteroposterior direction of HHM, our results show a significantly higher native and pathologic glenoid retroversion in case of posterior HHM. This is in accordance with previous research on shoulder OA, which showed a significantly higher native retroversion in patients with posterior HHM [9, 33] and a high correlation between HHM and pathologic glenoid version, both in amplitude and direction [31]. Secondly, we found that the anterior axis length was significantly larger in patients with posterior HHM. It is the distance between the coracoid and the intersection of the scapular plane with the fulcrum axis, which runs from the posterior acromion to the coracoid [22]. This indicates a more anteriorly translated coracoacromial arch relative to the scapular plane. These findings are in line with those in shoulder OA [33]. Finally, the influence of the RC on anteroposterior HHM in RCTA is not yet fully understood. The muscle Vol and FI of the transverse force couple are in equilibrium in non‐pathologic shoulders [16, 26]. The current hypothesis is that posterior HHM originates from the absence of the IS muscle combined with the posteriorly directed force of the latissimus dorsi [13]. Although we found a less voluminous posterior RC in all groups and subgroups of RCTA patients (Vol(IS/Ssc) < 1), there was a trend towards a higher Vol(IS/Ssc) in posterior HHM and an increase of Vol(IS/Ssc) was associated with an increase of posterior HHM. These findings seem to contradict the current hypothesis. To our knowledge, only one study investigated the influence of RC degeneration in RCTA on glenoid erosion [1], which correlates strongly with HHM when assessed in 3D [31]. They found that FI was more pronounced in the posterosuperior RC in RCTA patients with posterosuperior glenoid erosion. Contrastingly, an increase in Ssc FI was associated with an increase in posterior glenoid erosion. In shoulder OA, Aleem et al. found a higher IS/Ssc Vol in case of posterior HHM, which is in line with our findings [2]. Others found conflicting results [7, 29, 30, 37]. However, the aforementioned studies used 2D measurement techniques, which are less reliable [20, 30, 31]. Using a similar 3D technique as ours, Arenas‐Miquelez et al. found no difference in the IS/Ssc Vol between shoulder OA patients with and without posterior HHM [3]. They did find a higher FI ratio of IS/Ssc to be significantly associated with posterior HHM. They postulated that this was likely to be a consequence of posterior HHM, as posterior HHM causes a disturbed length–tension relationship with an increase in pennation angle and reduction of sarcomeres, which is shown to cause FI [11, 12, 21]. These contradictory findings seem to indicate that we have yet to discover the exact relationship between AP‐HHM and transverse force couple imbalance. It is clear, however, that bony scapular anatomy plays an important role in posterior HHM, as it can explain 31% of AP‐HHM variance.

The strength of the study is the use of highly accurate, state‐of‐the‐art 3D quantitative assessment of RC degeneration, scapular anatomy and HHM. 2D assessment of RC degeneration is most frequently used in other studies but is less accurate because the distribution of intra‐muscular fat is not uniform in the RC muscles [38] and because the cross‐sectional area of the RC muscle can be influenced by HHM [3]. Also, most previous studies used the Goutallier classification for assessing FI, which is limited due to its poor interobserver and intra‐observer reliability and its qualitative characteristics [20, 25]. Second, to our knowledge, this is the first study to incorporate RC degeneration, and native and pathologic scapular anatomy to investigate their association with HHM.

Nevertheless, this study has several limitations as well. First of all, we did not assess RC integrity. We opted to use FI and Vol of the RC muscles to account for their function, as they are shown to be directly related to force generation capacity [17, 26, 32]. Furthermore, FI and atrophy are shown to occur as a result of tendon injury [11, 12, 21]. Second, patient age and sex were not included in the analysis. Although FI is not influenced by age or sex in healthy shoulders, there is a significant difference in muscle Vol between males and females [16]. This may have caused less reliable results regarding the influence of RC atrophy on HHM. Thirdly, our data set was rather small, making our statistical analysis possibly underpowered to detect other significant associations. Finally, our models for explaining the variance of anteroposterior and superior HHM only reached 41% and 17%, respectively. Therefore, further research is mandatory and should include dynamic RC activation, other shoulder muscles (such as deltoid, latissimus dorsi and pectoralis major) [5] and scapulothoracic motion and position.

## CONCLUSION

In RCTA, degeneration of the posterosuperior RC and acromion morphology seems to be associated with superior HHM, while in the glenoid version, the rotational alignment of the coracoacromial complex and an imbalance in FI and muscle Vol in the transverse force couple seems to be associated with anteroposterior HHM.

## AUTHOR CONTRIBUTIONS

**Hannes E. Tytgat**: Analysis and interpretation of data/drafting the paper. **Nazanin Daneshvarhasjin**: Acquisition of data. **Philippe Debeer**: Approval of the final version. **Jean Chaoui**: Acquisition of data. **Filip Verhaegen**: Research design/critical revision of paper.

## CONFLICT OF INTEREST STATEMENT

The authors declare no conflicts of interest.

## ETHICS STATEMENT

This study was approved by the Ethics Committee Research UZ/KU Leuven (S58348). Informed consent was obtained from all individual participants included in the study.

## Supporting information

Supporting information.

Supporting information.

## Data Availability

The data that support the findings of this study are available from the corresponding author upon request.
